# Dendritic cell metabolism: immunity and tolerance

**DOI:** 10.18632/oncotarget.5865

**Published:** 2015-09-28

**Authors:** An-Sofie Vanherwegen, Conny Gysemans, Lut Overbergh

**Affiliations:** Laboratory of Clinical and Experimental Endocrinology, KU Leuven, Leuven, Belgium

**Keywords:** dendritic cells, vitamin D, tolerance, glycolysis

It becomes exceedingly clear that immune cells not only use cellular metabolism to meet their bioenergetic demands, but that these metabolic pathways also determine their phenotype and function. We now also acknowledge that perturbations of distinct metabolic networks are implicated in immune cell-associated diseases. “Immunometabolism” explores the metabolic machinery in all immune subsets and studies how it regulates their immunological function in either homeostatic or pathological situations. A distinct metabolic status between pro-inflammatory Ml and anti-inflammatory M2 macrophages has already been unraveled. Similar to the Warburg effect observed in cancer cells, M1 macrophages mainly use glucose consumption and lactate excretion, whereas M2 macrophages mostly rely on a combination of oxidative phosphorylation (OXPHOS), fatty acid oxidation (FAO) and mitochondrial respiration. In T lymphocytes, a similar shift in cellular metabolism could be observed in inflammatory T helper 17 (Thl7) cells, performing aerobic glycolysis and glutamine oxidation, and regulatory T cells (Treg), primarily depending on mitochondrial oxidative pathways to meet their energy needs [1]. Until recently, such perceptions on energy metabolism reprogramming were provocative as researchers assumed that cells would only rely on glycolysis when their mitochondria were impaired or oxygen was absent. Moreover, these hallmark studies highlighted the significance of cellular metabolism in the regulation of gene transcription and of metabolic enzymes in the control of protein translation and entire immunological processes such as differentiation and proliferation.

To date, metabolic reprogramming in dendritic cells (DCs) remains less documented. Immunogenic DCs initiate inflammation and prime antigen-specific effector T cell responses, whereas tolerogenic DCs induce and maintain T cell tolerance in the periphery [2]. This regulatory function of DCs is strictly reliant on their different stages of differentiation/maturation and activation. Based on these characteristics, DCs have become an appealing cell type for immunotherapy to either enhance inadequate immune responses in cancer or attenuate disproportionate immune responses in autoimmunity and chronic inflammation. Evidence is progressively accumulating that also DCs activated via their toll like receptor (TLR) switch from OXPHOS to glycolysis under aerobic conditions. This metabolic transition is promoted by the Akt pathway and inhibited by AMP-activated protein kinase (AMPK), a central regulator of catabolic pathways including β-oxidation of fatty acids. This metabolic signature which is active within minutes supports highly activated/energetic cells to fulfill biosynthetic pathways for the assembly of inflammatory surface and secretory molecules. Interestingly, intermediary metabolites of the tricarboxylic acid (TCA) cycle, such as citrate and succinate, also increase the immunogenicity of immune cells, even when TCA cycle activity is diminished [3].

Collectively, these observations suggest that therapeutic approaches that would dampen anabolic metabolism or glycolysis may promote the generation of immunogenic DCs, while the promotion of catabolic metabolism may support tolerogenic DC development. However, this view is over simplistic and a more nuanciated description is needed. High lactic acid concentration induces a potent tolerogenic reprogramming during DC differentiation [3]. 1,25-dihydroxyvitamin D_3_ (1,25(OH)_2_D_3_)-conditioned tolerogenic DCs convert glucose to lactate for fast, although less efficient, ATP generation, accompanying high glucose consumption, even when oxygen is available for OXPHOS. Transcriptomics and proteomics data further support the early metabolic reprogramming of 1,25(OH)_2_D_3_-conditioned tolerogenic DCs which is controlled by the PI3K/Akt/mTOR pathway [4, 5]. Also others documented a higher glycolytic capacity and reserve in tolerogenic DCs induced by 1,25(OH)_2_D_3_ in combination with dexamethasone [6]. Further insights in what causes these metabolic transitions and what their role is in directing the phenotypical switches is of great importance to identify new targets for drug development.

By manipulating metabolic pathways, immune cells can be re-educated in vivo either towards an immunogenic phenotype in case of cancer or towards a more tolerogenic phenotype to temper ongoing autoimmune activation. Immune cells are strongly influenced by nutrients present in the local environment. For example, highly glycolytic tumor cells consume all glucose in the tumor microenvironment also necessary for proper effector T cell differentiation. In addition, tumor-derived lactic acid blocks the cytotoxic T cell function. Therefore, manipulation of local metabolism can be a therapeutic approach to boost T cell activity [7]. Analogous strategies to modify the substrate availability at the inflammation site might be interesting to favor induction of tolerogenic DCs. However, stromal and epithelial cells, as well as other immune cells, are also present at the site of inflammation and share similar metabolic paths whereby they can be negatively influenced when DC glycolytic metabolism is enhanced. Therefore, antigen-specific immunotherapy with *ex* vivo 1,25(OH)_2_D_3_-conditioned DCs presents a promising clinical tool to restore tolerance in autoimmune diseases and transplantation. Moreover, the tolerogenic phenotype induced by 1,25(OH)_2_D_3_ is resistant to maturation and low oxygen concentrations, an essential feature as transferred DCs will home to inflammatory and hypoxic environments [2, 5]. Further metabolic insights will contribute to the development of DC-based immunotherapies.
